# Formation of biogenic amines in fish: Dietary intakes and health risk assessment

**DOI:** 10.1002/fsn3.2271

**Published:** 2021-04-06

**Authors:** Waleed Rizk El‐Ghareeb, Abdelazim Elsayed Elhelaly, Karima Mohamed Eissa Abdallah, Heba Mohamed M El‐Sherbiny, Wageh Sobhy Darwish

**Affiliations:** ^1^ Department of Public Health College of Veterinary Medicine King Faisal University Al‐Ahsa Saudi Arabia; ^2^ Department of Frontier Science for Imaging School of Medicine Gifu University Gifu Japan; ^3^ Department of Food Hygiene and Control Faculty of Veterinary Medicine Suez Canal University Ismailia Egypt; ^4^ Food Control Department Faculty of Veterinary Medicine Zagazig University Zagazig Egypt; ^5^ Faculty of Veterinary Medicine Educational Veterinary Hospital Mansoura University Mansoura Egypt

**Keywords:** biogenic amines, Egypt, Fish, microbial contamination, risk assessment

## Abstract

Biogenic amines (BAs) are toxicants that are produced during the proteolytic activities of some microorganisms, or naturally during the metabolism of their precursor amino acids. The objective of this study was to estimate the formed BAs in six types of fish retailed in Egypt including tilapia, mullet, mackerel, sardine, herring, and tuna. In addition, total mesophilic (TMC) and total psychrophilic (TPsC) bacterial counts were investigated. Furthermore, the estimated daily intakes (EDI) of BAs via the ingestion of various types of fish in Egypt were calculated, and their potential health risks were discussed. The achieved results indicated the formation of histamine (HIS), tyramine (TYR), cadaverine (CAD), putrescine, spermine, and spermidine at different concentrations. Tilapia had the lowest concentration levels for the different BAs. In contrast, mackerel and tuna had the highest concentrations of BAs. Total biogenic amines (TBAs) showed significant positive correlations with TMC in the examined fish species. The recorded EDI values of the different BAs in the current study would not have adverse effects, except for mackerel and tuna. Excessive consumption of fish contaminated with BA might have serious health hazards such as symptoms of histamine poisoning, including rashes, flushing, palpitations, and asthma. Therefore, the adoption of strict hygienic measures during the processing, storage, and distribution of fish is highly recommended to reduce the formation of BAs in fish.

## Graphical abstract

Tilapia had the lowest levels for the different tested biogenic amines. Mackerel and tuna had the highest concentrations of biogenic amines. Total biogenic amines showed significant positive correlations with total mesophilic counts in the examined fish species.

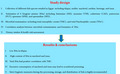

## 1 INTRODUCTION

Fish is considered as a major source for high biological values protein with low cholesterol, rich in trace elements and vitamins. In addition, fish is regarded as essential for a healthy life because of their content of n‐3 and n‐6 series of polyunsaturated fatty acids (PUFA), especially docosahexaenoic and eicosapentaenoic fatty acids **(**Brito et al., 2019; Lira et al., 2019, 2020 **)**. The demand for fish consumption is increasing worldwide to compensate the shortage of red meat. Fish is highly perishable protein source, and due to the improper hygienic and storage conditions during transportation, handling, and processing, microbial contamination and fish deterioration might occur leading to the formation of a vast array of chemicals **(**Huss, 1995 **)**.

Biogenic amines (BAs) are natural toxicants produced by a vast array of the microorganisms, either pathogenic or none, and via the metabolism of some amino acids. BAs are classified into 1) monoamines such as histamine (HIS) and tyramine (TYM) that are produced by a one‐step decarboxylation reaction from their precursor amino acid histidine and tyrosine, respectively (Marcobal et al., 2012 **)**. 2) Diamines such as cadaverine (CAD) which is produced by a decarboxylation reaction from lysine, and putrescine (PUT). The latter is a diamine that can be produced either by a single‐step decarboxylation from ornithine and agmatine or indirectly after hydrolysis of arginine **(**Wunderlichová et al., 2014 **)**. 3) Polyamines such as spermine (SPM) and spermidine (SPD) are produced by various pathways that include the hydrolysis of arginine into ornithine and agmatine **(**Shah & Swiatlo, 2008 **)**. Biogenic amines at their physiological concentrations play essential roles in several processes in the cell, including gene expression, cell growth, and tissue repair **(**Benkerroum, 2016; Galgano et al., 2012; Ma et al., 2020 **)**. Unlikely, ingestion of high levels of such BAs may lead to severe symptoms such as anaphylaxis, hypertensive symptoms, nervous manifestation, and death **(**Medina et al., 2003 **)**. In addition, some BAs such as PUT and CAD were associated with the onset of gastric cancer as it may be converted into carcinogenic N‐nitroso compounds by microorganisms in the digestive tract **(**Koutsoumanis et al., 2010 **)**. Ingestion of fish containing elevated levels of BAs was associated with cases of intoxication in Europe **(**EFSA Panel on Biological Hazards (BIOHAZ) (2011)**)**. Screening of the occurrence of BAs in fish is considered also as a good index for the fish quality and hygiene **(**Koutsoumanis et al., 2010; Visciano et al., 2012 **)**. There is a clear lack of information about the levels of BAs in different kinds of fish marketed in Egypt and their contribution to the estimated daily intakes of BAs among the Egyptian population. Furthermore, the correlation analysis between the microbial counts and the formation of BAs is less informed. Therefore, the objectives of the current study were firstly to estimate the formed BAs in six species commonly consumed in Egypt, namely, Tilapia, Mullet, Mackerel, Herrings, Sardine, and Tuna. Estimated daily intakes of BAs due to consumption of such fish kinds were additionally calculated among the Egyptian population. Furthermore, microbial counts including total plate counts (TPC) and total psychrophilic counts (TPsC) were investigated in the fish samples and their correlations with the total biogenic amines (TBAs) were analyzed.

## 2 MATERIALS AND METHODS

### 2.1 Fish samples

One hundred and twenty fish samples, including Nile Tilapia (*Oreochromis niloticus*), Mullet (*Mugil cephalus*), Mackerel (*Scomber scombrus Linnaeus*, 1758), Herrings (*Clupea harengus Linnaeus,* 1758), Sardine (*Sardinella aurita*), and Tuna (*Thunnus Albacures*) (*n* = 20 of each fish spp.) were collected randomly from local fish markets in Egypt. Fish samples were purchased on a daily basis in the morning and within 2 hr from the arrival of the fish into the market. Fish samples were intact, with a fresh smell, and reddish gills. Fish is usually preserved in ice to keep their internal temperature as low as possible. However, the fish sellers frequently move the fish with their uncleaned bared hands. The fish market is usually in the open air, and the fish is kept in wooden boxes, which are not frequently cleaned, and the chance of cross‐contamination is high. The collected samples were transferred cooled directly to the laboratory for the microbial examination and estimation of their BAs contents.

### 2.2 Content of biogenic amines

Ten grams from each sample were firstly homogenized with 100 ml of 10% trichloroacetic acid for 3 min at 17,608 g. Then, the homogenates were extracted in dark for one hour with shacking at 4ºC, followed by centrifugation for 20 min at 1,956 g, 4ºC. The supernatants were filtered through a Whatman filter No. 1 and the filtrates were kept at 4ºC until analysis. An amino acid analyzer (L‐8900, HITACHI, Japan) was used for quantitative estimation of BAs according to Kononiuk and Karwowska (2019). The amino acid analyzer was equipped with an Ostion LG AAA8 ion‐exchange column (3,6 × 100 8 µm). Gradient elution using Na+/K+ citric buffers was employed for separation. Colorimetric detection was done at 570 nm, after postcolumn derivatization of ninhydrin. The Contents of the biogenic amines including HIS, TYR, PUT, CAD, SPM, and SPD were determined with a reference to the amine standards (Merck KGaA, Darmstadt, Germany). The biogenic amine concentrations were reported as mg/kg fish.

### 2.3 Daily intake of BAs

The estimated human daily intake (EDI) for the quantified and total biogenic amines via consumption of fish in Egypt was calculated for Egyptian adults and children according to the following equation:

EDI =Ci × F_IR_ /BW

Where Ci is the concentration of individual or total BAs in the fish, and IR is the ingestion rate of fish in Egypt. F_IR_ is the fish ingestion rate in Egypt, which was estimated at 48.57 g/day (Food and Agricultural Organization (FAO), 2003); BW is the bodyweight, which was estimated at 70 kg for adults and 30 kg for children.

### 2.4 Determination of total microbial counts

The total plate (TPC) and total psychrophilic (TPsC) counts were screened according to the methods of American Public Health Association (APHA) (2001). In short, twenty‐five grams from each fish sample was homogenized in 225 ml of sterile buffered peptone water 0.1% at 2,500 rpm for 2 min. These homogenates represent the dilution of 10^–1^, and then decimal dilutions have proceeded. From each dilution, one ml was transferred into two sterile Petri dishes to which 15 ml of nutrient agar (Oxoid) were added. After thorough mixing and solidification, the inoculated plates were incubated at 37°C for 24 hr for TPC or incubation at 7ºC for ten days for TPsC. The agar plates having 30–300 pinpoint colonies were calculated and recorded as follow:

TPC or TPsC/g. = average No. of colonies ×reciprocal of the dilution.

Counted colonies expressed as log 10 cfu/g.

### 2.5 Statistical analysis

Measurements were carried out in duplicates and all values were expressed as means ± *SE*. Statistical analysis was evaluated using Tukey–Kramer HSD test. Pearson correlation analysis was done using the JMP statistical package, SAS Institute Inc. In all analyses, *p* < .05 was used to indicate statistical significance using the JMP statistical package, SAS Institute Inc.

## 3 RESULTS AND DISCUSSION

In the present study, six BAs were quantified in six kinds of fish marketed in Egypt. The formed individual and total BAs varied significantly based on the kind of the fish and their microbial status.

### 3.1 Contents of BAs in the examined fish spp

#### 3.1.1 Histamine

The obtained results revealed formation of HIS in all examined fish samples. All fish species contained levels of HIS lower than the maximum permissible limits (MPL) (100 mg/kg) (Food Drug Administration, 2011), except for Mackerel (90% of the examined samples), and Tuna (100% of the examined samples). Tuna had significantly (*p* < .05) the highest mean concentrations for HIS (217.900 ± 3.176 mg/kg), followed by Mackerel (110.800 ± 1.630 mg/kg), Sardine (78.550 ± 1.536 mg/kg), Herrings (39.350 ± 0.398 mg/kg), Mullet (22.550 ± 0.738 mg/kg), and Tilapia (17.700 ± 0.506 mg/kg), respectively (Table 1). One possible reason for the high content of HIS in Tuna and Mackerel is their high content of the free histidine reaching to 732–1460 mg/kg **(**Biji et al., 2016 **)**. The recorded concentrations of HIS in the current work were comparable to that recorded in Mackerel and Sardine marketed in Peru **(**Gonzaga et al., 2009 **)**, Egypt (Sabry et al., 2019), and Korea **(**Kang et al., 2019 **)**. Higher HIS levels (>300 mg/kg) were reported in Sardine from Serbia **(**Petrovic et al., 2016 **)**. While, lower HIS levels were recorded in Tilapia in a study conducted in Poland **(**Kulawik et al., 2013 **)**.

**TABLE 1:** Biogenic amines content (mg/kg) among the examined fish species

	Cadaverine		Histamine		Putrescine		Spermine		Spermidine		Tyramine	
	Mean	*SE*	Mean	*SE*	Mean	*SE*	Mean	*SE*	Mean	*SE*	Mean	*SE*
Tilapia	2.995	0.131^a^	17.700	0.506^a^	1.725	0.088^a^	2.115	0.051^a^	4.830	0.181^ab^	34.450	1.150^a^
Mullet	3.065	0.207^a^	22.550	0.738^a^	1.465	0.035^a^	1.394	0.050^b^	3.855	0.066^bc^	32.850	1.155^a^
Mackerel	210.050	3.374^b^	110.800	1.630^b^	30.600	1.276^b^	3.995	0.086^c^	5.485	0.133^a^	52.550	0.867^bc^
Herrings	6.172	0.073^a^	39.350	0.398^c^	5.975	0.207^a^	2.575	0.032^a^	3.450	0.059^c^	45.150	0.887^b^
Sardine	5.468	0.171^a^	78.550	1.536^d^	5.505	0.201^a^	3.365	0.068^c^	4.965	0.074^a^	60.100	0.856^c^
Tuna	156.200	4.512^c^	217.900	3.176^e^	169.400	9.492^c^	7.740	0.476^d^	15.400	0.359^d^	143.200	2.552^d^

Note

Values represent Means ± *SE* (*n* = 20/each).

Values in the same column carrying different superscript letters are significantly different at *p* < .05.

#### 3.1.2 Tyramine

TYR is detected in all examined samples in the present study with levels below the recommended MPL (100 mg/kg) **(**Silla Santos, 1996 **)**, except for Tuna which had all samples exceeding this limit. Tuna had the highest residues of TYR (143.200 ± 2.552 mg/kg), followed by Sardine (60.100 ± 0.856 mg/kg), Mackerel (52.550 ± 0.867 mg/kg), Herrings (45.150 ± 0.887 mg/kg), Tilapia (34.450 ± 1.150 mg/kg), and Mullet (32.850 ± 1.155 mg/kg), respectively (Table 1). TYR was detected at comparable levels in Herrings **(**Özogul et al., 2002 **)**, Sardine **(**Özogul & Özogul, 2006 **)**, Mackerel **(**Kim et al., 2009 **)**, canned Mackerel, and Sardine marketed in Ghana **(**Weremfo et al., 2020 **)**.

#### 3.1.3 Cadaverine

CAD is detected in all examined fish samples at concentrations below MPL (540 mg/kg) EFSA Panel on Biological Hazards (BIOHAZ) (2011). Tilapia had the lowest CAD residues (2.995 ± 0.131 mg/kg); while Mackerel had significantly (*p* < .05) the highest CAD levels (210.050 ± 3.374 mg/kg) (Table 1). Comparable levels of CAD were recorded in Herrings **(**Özogul et al., 2002 **)**, Sardine **(**Özogul & Özogul, 2006 **)**, Mackerel **(**Zhai et al., 2012 **)**, and Tilapia **(**Kulawik et al., 2013 **)**. However, higher CAD levels (863 mg/kg) were reported in the Indian Anchovy **(**Yongsawatdigul et al., 2004 **)**.

#### 3.1.4 Putrescine

Regarding PUT, the achieved results indicated the formation of PUT in all examined fish samples. Similar to CAD, Tuna had significantly the highest PUT residues. PUT levels ranged between 0.700–4.100 mg/kg in Tilapia, 1.110–2.400 mg/kg in Mullet, 15.000–44.000 mg/kg in Mackerel, 2.500–8.200 mg/kg in Herrings, 3.600–7.400 mg/kg in Sardine, and 112.000–211.000 mg/kg in Tuna (Table 1). The recorded PUT residues in the examined fish samples in the current investigation go in agreement with that recorded in Herrings **(**Özogul et al., 2002 **)**, Sardine **(**Özogul & Özogul, 2006 **)**, Mackerel **(**Zhai et al., 2012 **)**, and Tilapia **(**Kulawik et al., 2013 **)**. Relatively higher PUT levels (259.9 mg/kg) were reported in the Indian Anchovy **(**Yongsawatdigul et al., 2004 **)**.

#### 3.1.5 Spermine

SPM levels were estimated in the examined fish samples. Results showed that Tuna had the highest SPM content (7.740 ± 0.476 mg/kg); while Mullet had the lowest average concentrations (1.394 ± 0.050 mg/kg) (Table 1). Similarly, SPM was detected at 1.8 mg/kg in Mackerel **(**Zhai et al., 2012 **)**. Unlikely, SPM was not detected in Herrings **(**Özogul et al., 2002 **)**, and Sardine **(**Özogul & Özogul, 2006 **)**. Higher SPM levels were recorded in Tuna **(**Veciana‐Nogués et al., 1997 **)**, and Indian Anchovy **(**Yongsawatdigul et al., 2004 **)**.

#### 3.1.6 Spermidine

SPD is the other‐tested polyamine in the present study. The mean concentrations of SPD in the examined fish samples were as follows: Tuna (15.400 ± 0.359 mg/kg) > Mackerel (5.485 ± 0.133 mg/kg) > Sardine (4.965 ± 0.074 mg/kg) > Tilapia (4.830 ± 0.181 mg/kg) > Mullet (3.855 ± 0.066 mg/kg) > Herrings (3.450 ± 0.059 mg/kg) (Table 1). These concentrations agree with that recorded in Herrings **(**Özogul et al., 2002 **)**, Sardine **(**Özogul & Özogul, 2006 **)**, Mackerel **(**Zhai et al., 2012 **)**, and Tilapia **(**Kulawik et al., 2013 **)**.

### 3.2 Dietary intakes and health‐risk assessment of BAs

The EDI values (mg/kg BW/day) of HIS via consumption of different fish species in Egypt ranged between 0.012 in adults and 0.029 in children for Tilapia, and 0.151 in adults and 0.353 in children for Tuna (Table 2). Therefore, an adult of 70 kg BW might consume about 10.583 mg/day of HIS. Ingestion of fish containing higher levels of HIS is linked to scombroid poisoning (HIS‐intoxication), which is characterized by hot flushing, chest pain, nervous symptoms, cardiovascular symptoms, gastrointestinal troubles, and anaphylaxis FAO WHO (2012). EFSA Panel on biological hazards had established the no observed adverse effect level (NOAEL) of HIS at 50 mg EFSA Panel on Biological Hazards (BIOHAZ) (2011). This might indicate that the recorded EDI values of HIS in the current study would not have adverse effects on the human population. However, several outbreaks due to HIS‐intoxication were reported in Taiwan due to consumption of raw and processed fish **(**Chen et al., 2010 **)**. The Rapid Alert System for Food and Feed (RASFF) recorded 246 HIS notifications, of these 80 notifications were related to food poisoning cases in the European countries between 2005 and 2010 Rapid Alert System for Food and Feed (RASFF) (2010). Similarly, outbreaks of HIS‐intoxication were recorded in Australia from 2001 to 2013, mainly via consumption of Tuna **(**Knope et al., 2014 **)**. Furthermore, an outbreak of HIS‐intoxication was reported in children who consumed canned sardines in a kindergarten in Vojvodina province, northern Serbia **(**Petrovic et al., 2016 **)**. This reflects interindividual variation in their susceptibility to the onset of the symptoms of HIS poisoning. Such variation might be explained by the differences in the genetic predisposition, age, and consumption of other foodstuffs containing higher levels of HIS **(**Visciano et al., 2014 **)**. The calculated EDI values (mg/kg BW/day) for TYR ranged from 0.023 in adults and 0.053 in children for Mullet to 0.099 in adults and 0.232 in children for Tuna (Table 2). Paulsen et al., (2012) suggested a maximum tolerable level for TYR in Austria for high fish consumption at 950 mg/kg. This suggests that the recorded levels in the present study would not have adverse effects on the Egyptian population. However, TYR is a known vasoactive biogenic amine and is linked to hypertension crisis, increased heart rate, intracranial hemorrhage, and cardiac failure **(**Benkerroum, 2016 **)**. Therefore, great care is needed, particularly for the highly susceptible people. EDI values of CAD (mg/kg BW/day) ranged from 0.002 in adults and 0.005 in children for Tilapia to 0.146 in adults and 0.340 in children for Mackerel (Table 2). CAD may potentiate the toxicity of HIS if higher levels of CAD were ingested. In addition, CAD is associated with gastric and intestinal cancers **(**Benkerroum, 2016 **)**. EDI values of PUT (mg/kg BW/day) ranged from 0.001 in adults and 0.002 in children for Mullet to 0.118 in adults and 0.274 in children for Tuna (Table 2). There is less information available about NOAEL or the risk assessment of PUT due to their least‐toxic effects **(**Koutsoumanis et al., 2010 **)**. However, PUT may potentiate the toxic effects of the HIS and TYR via inhibition of their oxidative inhibition pathway **(**Bulushi et al., 2009 **)**. Besides, PUT is associated with the onset of neurodegenerative diseases and gastrointestinal cancers **(**Benkerroum, 2016 **)**. EDI values (mg/kg BW/day) for SPM in the tested fish samples ranged from 0.001 in adults and 0.002 in children for Mullet to 0.005 in adults and 0.125 in children for Tuna (Table 2). To the best of our knowledge, there is no available information on NOAEL for SPM in the fish. However, SPM is associated with several neurodegenerative diseases and neurological symptoms such as epilepsy, psoriasis, and cancer **(**Benkerroum, 2016 **)**. EDI values were calculated for SPD in the examined fish (Table 2). EDI values for SPD (mg/kg BW/day) 0.003 in adults and 0.008 in children for Tilapia, 0.003 in adults, and 0.006 in children for Mullet, 0.004 in adults and 0.009 in children for Mackerel, 0.002 in adults and 0.006 in children for Herrings, 0.003 in adults and 0.008 in children for Sardine, and 0.011 in adults and 0.025 in children for Tuna. Similar to SPM, there is no information about NOAEL for SPD in the fish. SPD is a precursor for the most potent carcinogenic N‐nitrosamines. Furthermore, SPD is associated with ischemia and cystic fibrosis and facilitates tumor progression **(**Benkerroum, 2016 **)**.

**TABLE 2:** Estimated daily intakes (mg/day/kg body weight) of biogenic amines via consumption of fish among the Egyptian population

	Tilapia		Mullet		Mackerel		Herrings		Sardine		Tuna	
	Adult	Children	Adult	Children	Adult	Children	Adult	Children	Adult	Children	Adult	Children
Cadaverine	0.002	0.005	0.002	0.005	0.146	0.340	0.004	0.009	0.004	0.009	0.108	0.253
Histamine	0.012	0.029	0.016	0.037	0.077	0.179	0.027	0.063	0.054	0.127	0.151	0.353
Putrescine	0.001	0.003	0.001	0.002	0.021	0.049	0.004	0.009	0.004	0.009	0.118	0.274
Spermine	0.001	0.003	0.001	0.002	0.003	0.006	0.002	0.004	0.002	0.005	0.005	0.125
Spermidine	0.003	0.008	0.003	0.006	0.004	0.009	0.002	0.006	0.003	0.008	0.011	0.025
Tyramine	0.024	0.056	0.023	0.053	0.036	0.085	0.031	0.073	0.042	0.097	0.099	0.232
Total BAs	0.044	0.103	0.045	0.106	0.287	0.669	0.071	0.166	0.109	0.256	0.493	1.149

### 3.3 Microbial counts and their correlation with total BAs in fish

Calculation of the total BAs among the examined fish species revealed that Tuna had the highest content of BAs (709.840 ± 10.554 mg/kg) > Mackerel (413.480 ± 5.344 mg/kg) > Sardine (157.952 ± 1.975 mg/kg) > Herrings (102.672 ± 1.358 mg/kg) > Mullet (65.179 ± 1.887 mg/kg) > Tilapia (63.815 ± 1.324 mg/kg), respectively (Table 3). The high level of total BAs in the scombroid fish species including Tuna and Mackerel is probably due to their high content of the respective‐free amino acids **(**Biji et al., 2016 **)**. In addition, Mackerel and Tuna are imported fish species in Egypt. Frequent freezing and thawing of the imported fish during storage, transportation, and handling with the increase in the surrounding temperature give rise to the microbial decomposition and rapid spoilage of the fish **(**Visciano et al., 2012 **)**. Therefore, we extended this study to investigate the microbial status of the examined fish species and the correlation analysis between the microbial counts (TPC and TPsC) and the total BAs. Interestingly, Tuna and Mackerel had the highest TPC (5.756 ± 0.086 and 5.757 ± 0.005 log 10 cfu/g, respectively), followed by Herrings (5.425 ± 0.005 log 10 cfu/g), Sardine (5.381 ± 0.013 log 10 cfu/g), Mullet (4.866 ± 0.016 log 10 cfu/g), and Tilapia (4.677 ± 0.012 log 10 cfu/g). TPsC was comparable to that of the TPC (Table 3). Nearly similar TPC counts were reported in Tilapia **(**Kulawik et al., 2013 **)**, and Tuna **(**Jääskeläinen et al., 2019 **)**. Interestingly, significant positive correlations were observed between TBAs and TPC in the different fish species, particularly in the Tilapia, Herrings, and Sardine (Table 3). Similarly, Positive correlations between microbial counts and the formation of BAs were reported in the fish **(**Visciano et al., 2012 **)**, camel meat **(**Tang et al., 2020 **)**, and cheese **(**Ma et al., 2020 **)**. These results highly suggest that the initial microbial counts, storage conditions, and the kind of the fish determine the extent of the formation of BAs in the raw fish. Therefore, it is extremely important to follow the industry guidelines during the processing and manufacture of fish including minimizing the microbial contamination of fish, proper handling of fish, and ensuring chilling temperature of no more than 4°C Food Drug Administration (2011).

**TABLE 3:** Total biogenic amines content, microbial counts, and their Pearson correlations in the examined fish species

Fish spp.	TBAs		TPC		TPsC		Pearson TBAs Vs TPC		Pearson TBAs Vs TPsC	
	Mean	*SE*	Mean	*SE*	Mean	*SE*	*r*	*p*	*r*	*p*
Tilapia	63.815	1.324^a^	4.677	0.012^a^	4.048	0.023^a^	**.599**	**.005**	**.846**	**<.00001**
Mullet	65.179	1.887^a^	4.866	0.016^b^	4.259	0.012^b^	−.264	.261	**.475**	**.034**
Mackerel	413.480	5.344^b^	5.757	0.005^c^	4.380	0.017^c^	.255	.277	.104	.662
Herrings	102.672	1.358^c^	5.425	0.005^d^	4.543	0.017^d^	**.625**	**.003**	.097	.684
Sardine	157.952	1.975^d^	5.381	0.013^d^	4.238	0.013^b^	**.576**	**.007**	.099	.677
Tuna	709.840	10.554^e^	5.756	0.086^d^	4.476	0.024^d^	.018	.939	.217	.358

Note

Abbreviations: TBAs: Total biogenic amines; TPC: Total plate counts; TPsC: Total psychrophilic counts.

Values in the same column carrying different superscript letters are significantly different at *p* < .05.

Values in bold are those that show positive and significant (*p* < .05) correlations.

## 4 CONCLUSION

The obtained results in the present study indicated detection of different BAs in the marketed fish in Egypt. Tuna and Mackerel had the highest total BAs and microbial counts. Calculation of EDI values of the tested BAs via ingestion of fish in Egypt revealed no potential risk to the Egyptian population. However, excessive consumption of fish contaminated with BAs, particularly HIS and TYR might cause serious health hazards, particularly scombroid poisoning. Therefore, efficient hygienic measures should be adopted during processing, storage, distribution, and marketing of fish in Egypt. Such hygienic measures include thorough washing and disinfection of the wooden boxes used during fish handling and storage, adequate personal hygiene, cleaning and disinfection of the floors, walls, and other surfaces of the fishery shops, proper storage, and preservation of fish. Furthermore, continuous monitoring for BAs formation in the other fish and shellfish species marketed in Egypt is highly recommended from both the food safety and public health point of view.

## 5 COMPLIANCE WITH ETHICAL STANDARDS

### 5.1 Ethical approval

This study does not involve any human or animal testing. This study was conducted according to the guidelines of King Faisal University, Saudi Arabia.

### 5.2 Consent to participate

All authors approved to participate in this research work and in the manuscript.

### 5.3 Consent to publish

All authors approved this manuscript to be published.

## CONFLICT OF INTEREST

The authors declare that they have no conflict of interest.

## AUTHOR CONTRIBUTIONS

All authors contributed to the study conception and design. Material preparation and sample collections were performed by Waleed Rizk El‐Ghareeb and Karima Mohamed Eissa. Wageh Sobhy Darwish, Waleed Rizk El‐Ghareeb and Abdelazim Elsayed Elhelaly conducted chemical analysis and data managements. Wageh Sobhy Darwish, Heba Mohamed M. El‐Sherbiny, and Waleed Rizk El‐Ghareeb wrote the first draft of the manuscript. All authors commented on the previous versions of the manuscript and approved the final version before submission.

## Data Availability

All data and materials will be available upon request.
